# Lime Juice and Vinegar Injections as a Cheap and Natural Alternative to Control COTS Outbreaks

**DOI:** 10.1371/journal.pone.0137605

**Published:** 2015-09-10

**Authors:** Grégoire Moutardier, Sompert Gereva, Suzanne C. Mills, Mehdi Adjeroud, Ricardo Beldade, Jayven Ham, Rocky Kaku, Pascal Dumas

**Affiliations:** 1 IRD, UMR 9220 ENTROPIE, BP A5, Nouméa, New Caledonia; 2 Fisheries Department of Vanuatu, PMB 9045, Port-Vila, Vanuatu; 3 Laboratoire d’Excellence “Corail”, 58 Avenue Paul Alduy, 66860, Perpignan CEDEX, France; 4 CRIOBE, USR 3278 CNRS - EPHE - UPVD, 52 Avenue Paul Alduy, 66860, Perpignan, France; 5 MARE - Marine and Environmental Sciences Centre, Faculdade de Ciências da Universidade de Lisboa, Campo Grande, 1749–016, Lisboa, Portugal; 6 IRD, UMR 9220 ENTROPIE, Université de Perpignan, 52 Avenue Paul Alduy, 66860, Perpignan, France; Seagrass Ecosystem Research Group, Swansea University, UNITED KINGDOM

## Abstract

Outbreaks of the corallivorous crown-of-thorns seastar *Acanthaster planci* (COTS) represent one of the greatest disturbances to coral reef ecosystems in the Indo-Pacific, affecting not only coral reefs but also the coastal communities which rely on their resources. While injection approaches are increasingly used in an attempt to control COTS densities, most of them display severe drawbacks including logistical challenges, high residual environmental impacts or low cost-effectiveness. We tested a new alternative control method based upon acidic injections of cheap, 100% natural products. We investigated the lethal doses, intra- and inter-specific disease transmission and immune responses of COTS when injected with fresh lime juice (extracted from local *Citrus arantifolia)* and white spirit vinegar. High COTS mortality was achieved with small volumes: 10–20 ml per seastar induced death in 89%/97% of injected specimens after an average 34.3 h/29.8 h for lime juice and vinegar respectively. Highest efficiency was reached for both solutions with double shots of (2 × 10 ml) in two different areas on the body: 100% mortality occurred within 12–24 h, which is similar or faster compared with other current injection methods. Multiple immune measures suggested that death was very likely caused by pH stress from the acidic solutions rather than a bacterial infection. Contagion to either conspecifics or a variety of other reef species was not observed, even at COTS densities 15 times higher than the highest naturally reported. 10 to 20 l lime juice/vinegar could kill up to a thousand COTS at a cost of less than 0.05 USD per specimen; no permits or special handling procedures are required. We conclude that injections of lime juice and vinegar offer great advantages when compared to current best practises and constitute a cheap and natural option for all reefs affected by COTS.

## Introduction

At low density, the corallivorous crown-of-thorns seastar (COTS), *Acanthaster planci* (Linnaeus 1758) is an integral part of coral reef ecosystems. Yet, population outbreaks of this species represent the most severe biological disturbance experienced by coral reefs across the Indo-Pacific, from the coast of South Africa to the Gulf of California [1–5]. Outbreaks of COTS cause widespread damage to reef-building corals [6, 7] and the cascading effects from coral loss can severely harm the entire coral community [8–11]. In most Pacific countries where local people depend upon coral reefs for their livelihood, COTS constitute a recurrent threat to food security and the coastal communities’ lifestyle. There is historical evidence that coral reefs can recover from COTS outbreaks; however, given the current widespread declines in coral cover, they drive even more pressure on already weakened systems [12–13]. For example, it is predicted that the absence of COTS alone would reverse the currently declining curve of coral cover on the Great Barrier Reef [14]. Furthermore, the frequency of COTS outbreaks has been increasing over recent decades [15–17] and the outbreaks themselves are reaching record-breaking levels (e.g. 53 750 ind.km^-2^ [18]).

Currently, the impacts of COTS outbreaks can only be limited through direct human intervention. While numerous approaches have been developed over the last few decades (see review [19]), manual collection followed by disposal ashore is the most common technique used across the Pacific. It is one of the most robust methods to regulate COTS outbreaks, at least on a small scale [20]. Nevertheless, hand-removal may not be appropriate for severe outbreaks and/or large affected areas, as it requires significant manpower, long-term commitment and entails a high risk of injury for the participants. Injection approaches—where COTS are injected with a variety of noxious solutions—are increasingly used, as they are highly cost-effective and fairly safe when handled correctly [21]. However, they also include drawbacks: most solutions injected over recent decades were not only noxious for COTS but for the coral community as well. For example, formalin, ammonium hydroxide and sodium bisulphate were repeatedly used in Micronesia in the 70’s in an attempt to control COTS [22, 23, 24]. Yet, injections with sodium bisulphate are required at such high concentrations that they can lower oxygen levels in seawater [25, 26]. Sodium hypochlorite, ammonium hydroxide, copper sulphate and ammonia were used on the Great Barrier Reef until they were judged potentially toxic to fish and many invertebrates [27, 28]. Other solutions may favour bacterial induction and the growth of a particular type of pathogen [29]. As a cultural medium for vibrios, thiosulfate—citrate—bile—sucrose agar (TCBS) was found to induce disease and ultimately death in COTS, but with potential knock-on effects on the coral-associated community [30, 31]. Recently, single injections of 10 ml of TCBS protein ingredients (oxbile and oxgall) induced a strong immune response and death in COTS with no evidence of negative impacts on the coral community, and were considered a promising alternative control method [32].

Nevertheless, the costs of the current injection methods are out of the reach for many stakeholders. The price of injections may reach circa 35 USD per seastar (e.g. copper sulphate [33]), preventing countries with insufficient resources from developing efficient COTS control programmes. Even the alternative oxbile and bile salts injection method proposed by Rivera-Posada et al. [32] may remain out of reach financially for most coastal communities. In some Pacific countries, the cost of importing 250 g container(s) of oxbile exceeds 900 USD, freight cost included. Against this backdrop, developing more cost-effective approaches is critical.

In this paper we investigate an innovative method of controlling COTS outbreaks with injections of natural acidic solutions: lime juice and vinegar. Acetic acid, the active component of white vinegar, has previously been found to be lethal to COTS [34, 35]. However, these findings were based on limited, unrepeated experiments and potential impacts on other organisms via horizontal transmission were not tested. Lime juice extracted from limes, *Citrus aurantifolia*, contains a high percentage of citric acid and has never been tested on COTS. Both are common, inexpensive acidic agents that can be widely found in the Pacific region and do not require permits or special handling procedures.

This work was conducted in Vanuatu, a small archipelago in the Western Pacific where the presence of COTS has frequently been reported in recent decades [36–40]. The objectives were to 1) establish the effects of injecting both different acidic solutions and different volumes (including single *vs*. double shots) on the mortality rate and time to death of COTS under aquaria and field conditions, 2) to assess the potential side-effects of injections by testing intra- and inter-specific transmission on a range of reef-associated species, and 3) to investigate intra-cellular reactions and immune responses of COTS after injections. Our ultimate goal is to propose a highly efficient, affordable, and less harmful alternative compared to the control methods currently in use in the Pacific (sodium bisulphate, TCBS, oxbile and bile salts).

## Materials and Methods

### Study location—collection of test specimens

Experiments were conducted from February 2014 to September 2014 at the marine hatchery and aquaculture facilities of the Fisheries Department of Port-Vila, Vanuatu. Specimens of COTS were collected from the shallow fringing reefs in the surrounding islands of Moso (17°31’S, 168°15’E), Lelepa (17°34’S, 168°12’E) and Emao (17°29’S, 168°30’E) after permission was obtained from the customary land owners. In the field, COTS were manually collected by snorkelers; specimens damaged during collection were discarded and not used for the experiments. COTS were transported to the marine hatchery in 100 l containers filled with seawater regularly renewed, then maintained in large concrete tanks (5.00 m × 1.50 m × 0.64 m; length × breadth × height) with constant filtered seawater inflow for seven days prior to trials. *In situ* experiments were conducted on the island of Aore (15°35’S, 167°10’E), Vanuatu. Forty COTS were collected from two different fringing reef sites, located on the south-east (15°36’38.76”S, 167°12’16.14”E) and the south west coast of the island (15°36’36.48”S, 167°0.9’51.30”E).

### Acidic injection trials

Lime juice was extracted from the fruit of *Citrus aurantifolia* and filtered twice prior to use (average pH 1.8). White spirit vinegar (“cooking quality”, 8%) was obtained from local stores and used undiluted (average pH 2.2).

### Aquaria injection trials

Eight injection treatments were tested: four treatments with injections in one single area (single shots) and four treatments with injections in two opposite areas on the seastar (double shots). For lime juice, we tested three different single-shot volumes: 10, 15 and 20 ml and two double-shot volumes of (2×5 ml) and (2×10 ml). For vinegar, we tested a single-shot volume of 15 ml and two double-shot volumes of (2×5 ml) and (2×10 ml).

Experimental blocks consisted of eight individual aquaria (60 l) with constant filtered seawater inflow (27.5°C, pH = 7.8) each containing only one COTS. The specimens of COTS were randomly assigned to either control (no injection; three individuals) or acidic injection (five individuals) treatment per trial. We carried out four replicate trials for each treatment, such that 32 individuals were used for each of the eight injection treatments, i.e. 256 COTS were used in total (eight treatments × four replicate trials × eight COTS per trial). Specimens were injected in the arm junction using disposable syringes; for double-shot treatments, injections were performed in two opposite arm junctions. Each individual was monitored every 12 h for four days for clinical signs of stress such as mucus production, necrosis and loss of limbs; death was identified by the absence of reaction or movement from the podia [18, 32]. We recorded mortality rate per trial (% of dead specimens) and time to death for each individual.

### Field trials

Due to unfavourable field conditions, only one experimental trial was carried out. Based upon the results of the aquaria trials, we tested the double shot (2×10 ml) injections of lime juice. Experimental blocks consisted of two plastic cages (4 m^2^, 1 cm mesh size) anchored to the reef substrate. Specimens were randomly assigned to either control (no injection; *n* = 20) or acidic injection (*n* = 20) treatment. COTS were injected and monitored using the same protocol as for the aquaria experiment.

### Contagion trials

A contagion experiment was carried out to investigate the presence of intra- and inter-species transmission of disease or other noxious substances linked to our injection method. Experimental blocks consisted of 3 large concrete tanks (5.00 m × 1.50 m × 0.64 m; length × breadth × height) with constant filtered seawater inflow containing six COTS and a total of 30 other coral reef organisms. The 30 specimens belonged to phylums Cnidaria, Echinodermata, Mollusca and Chordata (Table 1) and had been collected from a healthy, shallow fringing reef located near the marine hatchery. Coral samples were placed in cages in order to prevent direct feeding injuries from COTS. Three temporal contagion trials were performed: after a seven day acclimation period, the six COTS specimens were randomly assigned to either no injection (three individuals) or a single-shot injection of 10 ml of lime juice (three individuals). The injection volume was chosen in order to avoid massive COTS mortality at the beginning of the trial so that we would be able to investigate the long-term effects of contagion. The trials ran for seven days after injection; specimens were monitored every 12h using the same protocol as for the aquaria experiment. The decaying/dead COTS were not removed from the tanks until the end of the experiment. An additional control trial (where none of the six COTS were injected) was simultaneously carried out.

**Table 1:** List of organisms used in the contagion experiments.

Taxonomic groups			Number of individuals			
Phylum	Class	Species	T1	T2	T3	Control
Cnidaria	Anthozoa	*Acropora sp*.	1	1	1	2
Cnidaria	Anthozoa	*Porites lobata* (Dana, 1846)	1	1	1	1
Cnidaria	Anthozoa	*Porites cylindrica* (Dana, 1846)	1	1	0	1
Cnidaria	Hydrozoa	*Millepora sp*.	0	0	1	1
Echinodermata	Echinoidea	*Diadema setosum* (Leske, 1778)	4	4	4	4
Echinodermata	Echinoidea	*Tripneustes gratilla* (Müller & Troschel, 1842)	1	1	1	1
Echinodermata	Echinoidea	*Mespilia globules* (Linneaus, 1758)	1	1	1	1
Echinodermata	Echinoidea	*Culcita novaeguineae* (Linneaus, 1758)	2	1	2	2
Echinodermata	Asteroidea	*Linckia laevigata* (Gray, 1840)	3	3	3	4
Echinodermata	Asteroidea	*Archaster typicus* (Müller & Troschel, 1840)	3	3	3	6
Echinodermata	Asteroidea	*Protoreaster nodosus* (Linneaus, 1758)	2	2	2	3
Echinodermata	Holothuroidea	*Holothuria atra* (Jäger, 1833)	3	3	3	4
Echinodermata	Holothuroidea	*Stichopus herrmanni* (Semper, 1868)	0	1	0	1
Echinodermata	Holothuroidea	*Holothuria hilla* (Lesson, 1830)	1	1	1	1
Mollusca	Gastropoda	*Trochus niloticus* (Linneaus, 1767)	6	6	6	6
Chordata	Actinopterygii	*Naso vlamingii* (Valenciennes, 1835)	1	1	0	1

### Mechanistic basis for death from acidic solutions

In order to determine the mechanism triggering death after acidic injections, we analysed different measures of the immune system of COTS. Immune responses were measured before and after four injection treatments. Two consisted of control injections (no injection or a single-shot injection of 10 ml of artificial seawater), and two consisted of acidic injections (single-shot injections of 10 ml of vinegar or 10 ml of lime juice). Experimental blocks consisted of four large concrete tanks (5.00 m × 1.50 m × 0.64 m; length × breadth × height) continuously supplied with fresh seawater and four plastic cages (4 m^2^; 1 cm mesh size) anchored to the reef substrate in the lagoon. Two trials were carried out over a period of 3 weeks. In the first, seventy-five COTS were randomly assigned to one of three treatments: no injection (*n* = 25), artificial seawater injection (*n* = 25) or lime juice injection (*n* = 25). In the second trial, thirty COTS were randomly assigned to one of two treatments: artificial seawater injection (*n* = 15) or vinegar injection (*n* = 15).

### Baseline (pre-treatment) immune response

Within three hours of collection from the fringing reef, 1.5 ml of coelomic fluid (CF) was withdrawn into an equal volume of cold anti-aggregative solution (AG) from a subset of COTS according to the methods described in Mills ([41]). The CF-AG suspension was then subdivided into three sub-samples in order to measure three aspects of pre-treatment immune function described below.

### Post-treatment immune response

*Micrococcus lysodeikticus* was injected into each COTS (approx. 0.06 ml per arm, [42]) 5.5 days prior to each injection treatment to simulate bacterial invasion. 6 days post-bacterial challenge and 12 h post-injection treatment, CF was collected and sub-divided into three sub-samples as described above. Immediately after CF collection, wet weight was measured for each individual and their sex determined from gonad smears.

### Measures of immune function

All the methods were adapted for COTS as described in Mills ([41]); further details are given in S1 File.

#### Lysosomal membrane integrity

The loss of a neutral red dye from lysosomes into the cytosol was used as a measure of lysosomal membrane integrity, an indicator of amoebocyte cell viability [43–45].

#### Oxygen metabolism

The reduction of nitroblue tetrazolium by phagocytosing coelomcytes mediated through superoxide anions (O_2_^−^) was used as a measure of microbicidal activity.

#### Peroxidase activity

We used an immunoenzymatic analysis as a measure of the amount of peroxidase released during degranulation as an indicator of the activity of antimicrobial cytotoxic molecules. Baseline measures of peroxidase activity were not taken in the second trial due to a limiting amount of sulphuric acid that was preferentially conserved for post-treatment measures.

### Statistical analysis

#### Mortality rate

As data could not be satisfactorily normalised, differences in mortality rates between treatments were compared for each injection solution separately using Kruskall-Wallis H tests. A Mann-Whitney U test was used to determine the effect of the solution injected (lime juice *vs*. vinegar) on mortality rate, for both of the double-shot volumes (2x5 ml and 2x10 ml) used in the trials.

#### Time to death

The effects of injection treatments on time to death were tested using ANCOVAs with Tukey’s HSD post-hoc tests when applicable, after data were successfully log-transformed to meet the assumption of normality. The effects of treatment (fixed factor) were tested for lime juice and vinegar separately while controlling for the random effects of diameter and number of arms. Correlation between size and time to death was further investigated using Pearson’s correlation moment. Student’s t Tests were used to compare the time to death between the acidic solution double-shot injections carried out in aquaria, and between the aquaria and the *in situ* experiments for 10ml injections of lime juice.

#### Immune responses

ANOVAs were carried out on the three baseline immune measures, to test for the effects of treatment (fixed effect) while controlling for the random effect of aquarium. ANCOVAs were carried out on the three post-injection immune measures to test for the effects of treatment (fixed effect) while controlling for the random effect of aquarium, and using sex and wet weight as covariates. All the analyses were carried out after log or reciprocal transformation, to meet the assumption of normality where necessary. Statistical analyses were conducted using R software and Statistica 10 package.

## Results

### Testing injections of acidic solutions

#### Aquaria injection trials

All treatments induced signs of decay 6–12 h post-injection. The characteristic clinical signs showed by injected specimens included mucus production, ulcerations, matting/loss of spines, loss of body turgor and necrosis of the skin usually starting along the closest arms to the injection point. If alive, loss of limbs occurred in some cases as early as 12 h after injection. Regardless of the quantity used, all of the injected specimens had lost at least one third of their body at the end of the experiment (four days). On the other hand, only three out of the 96 control specimens exhibited some signs of decay during the experiments.

#### Mortality rate

**Fig 1:**
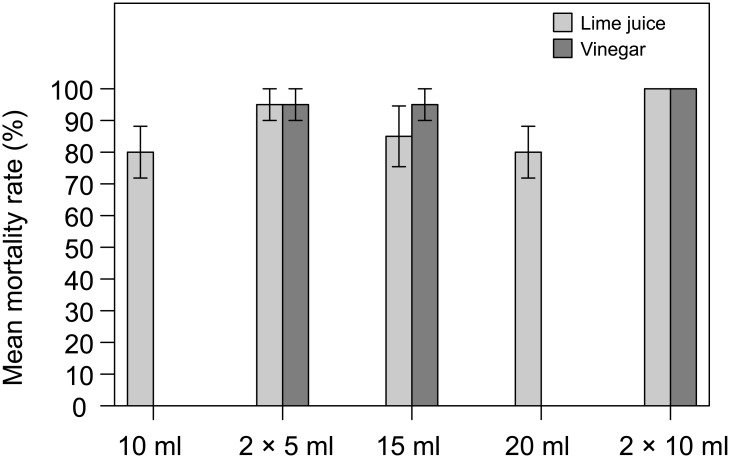
Mortality rates for COTS subjected to different injection treatments. Means ± SE for lime juice (light grey) and vinegar (dark grey).

High mortality was observed regardless of the solution or volume injected. Average mortality ranged from 80 to 100% (mean 89%) and from 95 to 100% (mean 97%) for lime juice and vinegar respectively (Fig 1). No significant differences were found in mortality rate between the different treatments (Kruskall-Wallis tests; lime juice: χ^2^(4) = 6.21, *p* = 0.18; vinegar χ^2^(2) = 1.1, *p* = 0.58). Mortality rate was similar for lime juice and vinegar regardless of double-shot volume (Mann-Whitney U tests; 2×5 ml: *U* = 12, *n* = 12, *p* = 0.49; 2×10 ml: *U* = 10, *n* = 12, *p* = 0.22).

#### Time to death

**Fig 2:**
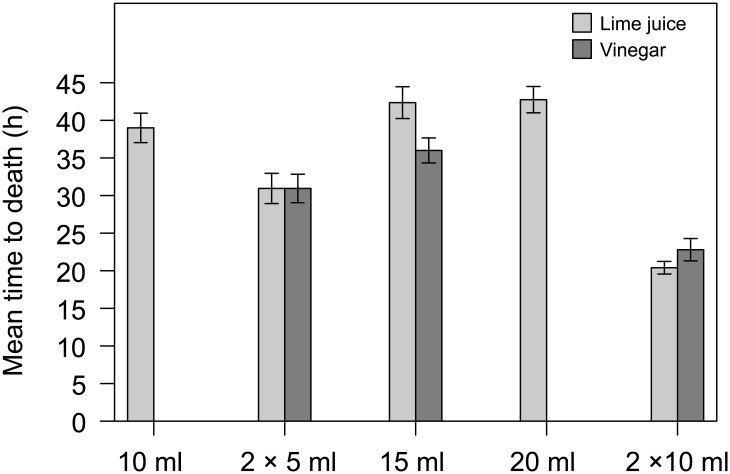
Time to death for COTS subjected to different injection treatments. Means ± SE for lime juice (light grey) and vinegar (dark grey).

Lime juice injections began to induce death after as little as 12h, with average time to death being 34.3h (Fig 2). While increasing the volume from 10ml to 20ml had no significant effect on time to death for COTS injected with single shots, double-shot injections of 2×10 ml induced death more quickly than any other treatment (ANCOVA, treatment: *F*_4,78_ = 6.98, *p* < 0.001; post hoc tests 2×10 ml versus all others, *p* < 0.01) (Fig 2). All but one of the specimens injected with 2×10 ml lime juice died within 12–24 h after injection and average time to death was 20.1 h. Time to death was not influenced by the number of arms nor by the diameter of the specimens (ANCOVA, arms: *F*_1,78_ = 0.45, *p* = 0.50; diameter: *F*_1,78_ = 0.24, *p* = 0.62).

COTS injected with vinegar died after 29.8 h on average (minimum 12 h) (Fig 2); no significant differences in time to death were observed among the different treatments (ANCOVA, treatment: *F*_2,52_ = 2.14, *p* = 0.13). Time to death, however, increased significantly with the diameter of the specimen (ANCOVA, diameter: *F*_1,52_ = 17.87, *p* < 0.001; Pearson’s correlation, *r* = 0.48, *n* = 57, *p* < 0.001). Time to death was similar for both lime juice and vinegar for 2×10 ml double shots (Student’s t Test; t(38) = -0.20, *p* = 0.84).

#### Field trial

**Fig 3:**
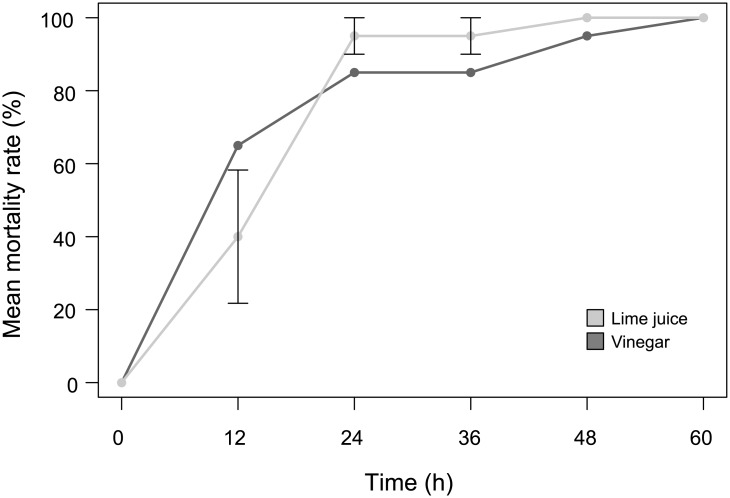
Temporal evolution of the mortality rate for COTS injected with 2×10 ml of lime juice. Aquaria (light grey; means ± SE) *vs*. *in situ* (dark grey) trials.

Injections of lime juice (2×10 ml) induced death in all COTS (Fig 3). Mucus, severe necrosis and loss of limbs appeared within 12 h post-injections. While 17 out of the 20 specimens died within the first 24 h, the remaining three lasted longer (up to 2.5 days); death occurred after 20.4 h on average. None of the controls died or exhibited any signs of decay. The COTS injected *in situ* responded the same way as in the aquaria experiments (Fig 3). No significant differences were observed in time to death between trials conducted *in situ* versus those in aquaria conditions (Student’s t Test for 2×10 ml lime juice injections; t(38) = 0.49, *p* = 0.63).

#### Contagion trials

All but two COTS injected with 10 ml lime juice (single shot) died; mortality ranged from 67 to 100% (average 78%) during the trials. The two COTS that remained alive at the end of the trial period exhibited severe necrosis. Non-injected COTS were not affected by the presence of injected conspecifics, and presented no signs of reaction or clinical signs of decay including mucus, necrosis or loss of limbs during the trials.

Only two of the 90 non-COTS specimens used for the contagion trials showed signs of disease. One specimen of seastar *Linckia laevigata* was affected by a minor, 1.5 cm necrosis on one arm after six days; and a coral colony of *Acropora sp*. partially bleached on the 2^nd^ day. None of the other specimens tested showed any evidence of physical or behavioural symptoms of decay.

### Mechanistic basis for death from acidic solutions

#### Baseline (pre-treatment) immune measures

**Fig 4:**
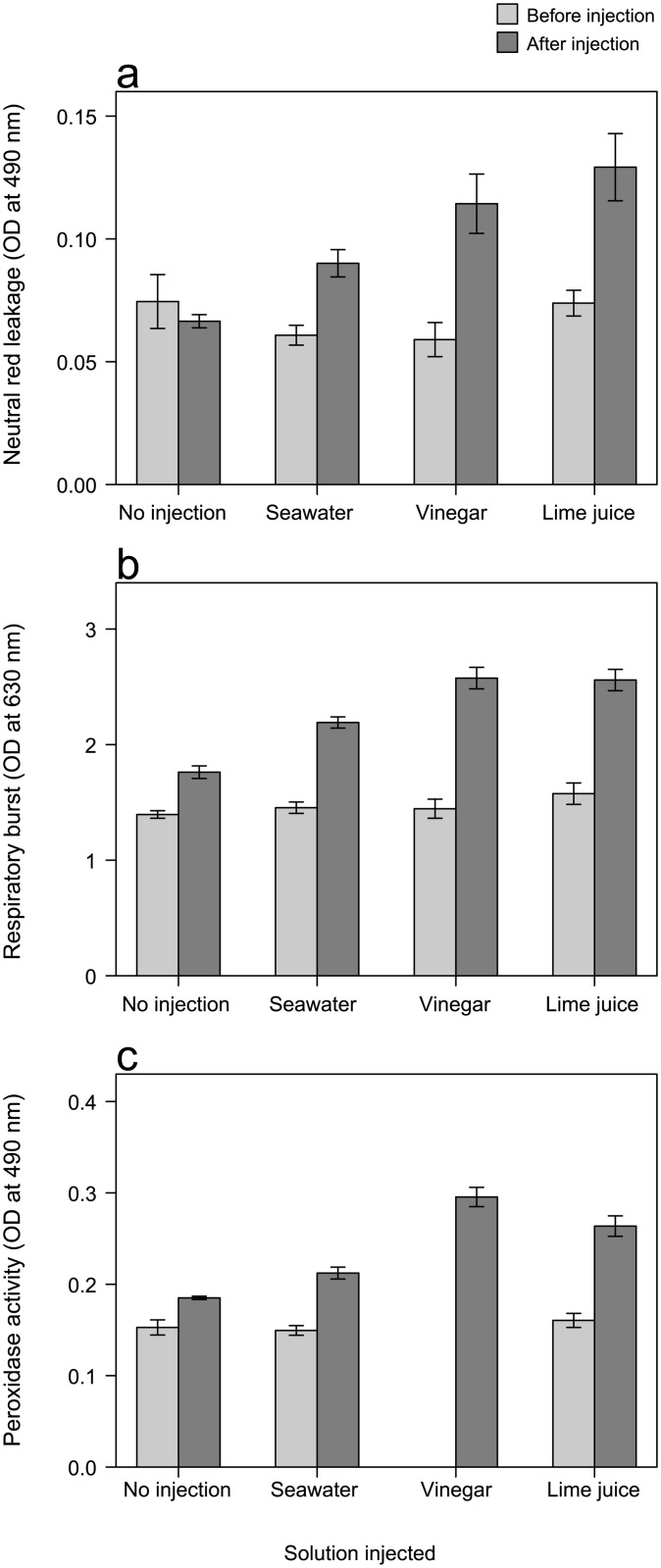
Results of three immune measures before (light grey) and after (dark grey) injection treatments. Means (±SE) for **a** neutral red leakage into cytosol of the cell (standardised optical density) as a measure of lysosomal membrane integrity of amoebocytes, **b** reactive oxygen species production (standardised optical density) as a measure of respiratory burst and **c** peroxidase activity (standardised optical density).

No differences were found in any pre-treatment immune measure between the four injection treatments (ANOVA; Lysosomal integrity: *F*_3,71_ = 1.181, *p* = 0.323; Respiratory burst: *F*_3,71_ = 0.654, *p* = 0.583; Peroxidase activity: *F*_2,45_ = 2.212, *p* = 0.121) (Fig 4).

#### Post-treatment immune measures

In contrast to pre-treatment results, significant differences between the four treatments were found for all of the immune measures (ANCOVA; Lysosomal integrity: *F*_3,67_ = 9.945, *p* < 0.001; Respiratory burst: *F*_3,65_ = 15.345, *p* < 0.001; Peroxidase activity: *F*_3,64_ = 22.803, *p* < 0.001) (Fig 4). Post Hoc tests revealed that all three immune responses of COTS injected with either lime juice or vinegar were significantly higher than those of both non-injected COTS (all *p* ≤ 0.001) and COTS injected with artificial seawater (all *p* < 0.02), except for the measure of lysosomal integrity after injection with vinegar (*p* = 0.192). The immune responses of COTS injected with artificial seawater were also higher than non-injected COTS (all *p* < 0.02) except for peroxidase activity (*p* = 0.398). However, no differences were found between the immune responses of COTS injected with lime juice and vinegar (lysosomal integrity: *p* = 0.357; respiratory burst: *p* = 0.861) except for peroxidase activity which was higher for vinegar (*p* = 0.031).

## Discussion

### Responses of COTS to acidic injections

It is now widely recognised that attempts to control COTS outbreaks have been largely unsuccessful, despite several decades of research and significant financial commitment [19]. In this paper we tested a new alternative control method based upon acidic injections of cheap, 100% natural products, widely-available and requiring no permits or special handling procedures. Our results highlight that high COTS mortality is achieved with small volumes (10–20 ml per seastar) of both acidic solutions tested. Whether the COTS were injected with lime juice or vinegar made almost no difference. Double-shot injections of 10 ml located in the junction area of two opposite arms were the most efficient: death was induced in all the injected seastars within 1 day, under both experimental and field conditions. Reducing the quantity by half (double-shots of 5 ml per seastar) was also shown to be an effective, albeit slower method of inducing death. Here, 95% mortality was achieved, while the remaining 5% specimens suffered from several necroses and lost at least one third of their body. Moreover, in the natural environment these severely damaged COTS are likely to constitute attractive prey for opportunistic predators including a variety of puffer fish or triggerfish species [46], the small painted shrimp (*Hymenocera picta* [47]) or some polychaete worms (e.g. *Pherecardia striata* [48]), among others.

Under double shots of 10 ml of either lime juice or vinegar, death occurred in less than 24h, which is similar or shorter than with other current injection methods. Thiosulfate—citrate—bile—sucrose agar (TCBS) or some of its components (peptone, oxgall, bile salts) were found inducing death within 24-48h, depending on the concentration and temperature [21, 31]. Similarly, injections of hypersaline solutions (145–345 ppt) caused 50–99% mortality in COTS after 24h [49, 50]. Moreover, while minimising the risk of damaging corals, double shots are considered safer and more time-effective than methods requiring multiple shots to reach similar death rates, e.g. 5 injections per COTS with hypersaline solutions and up to 25 injections when sodium bisulphate is used [50, 51].

### Cellular mechanisms triggering COTS death

We aimed to determine the cause of death of the COTS injected with acidic solutions. Death may have been induced via pH stress caused by the low pH of the injections altering various physiological mechanisms and causing failure of the immune system and reproductive functions [52]. Alternatively, the injections may have either induced a transmissible bacterial disease or weakened the immune system and caused a secondary bacterial infection due to opportunistic pathogens. Contagion to conspecifics was not observed, despite COTS densities 15 times higher than those usually reported from coral reefs (0.89 COTS m^-2^ in this study *vs*. 0.05 COTS m^-2^ [18]). For non-COTS organisms, only 2 out of the 30 specimens showed very minor signs of disease that did not induce serious injuries or death. Moreover, this occurred at the very beginning of the experiments, and was more likely the consequence of transplantation stress. This happened in particular with other colonies of *Acropora* sp. that showed similar symptoms during the acclimation phase, and thus were discarded before the start of the contagion trials. Therefore, whilst acidic solution injections inflicted death on COTS, we conclude that no disease transmission to other COTS, or other reef organisms could be observed.

One of the immune responses measured involves lysosomal membrane stability. Lysosomes degrade macromolecules, a membrane-dependent process, thus the stability of the lysosomal membrane is essential for detoxification and defence in marine organisms [43, 44]. Neutral red passively diffuses through the cell membrane [53], however, its retention within the lysosome depends on lysosome pH and its membrane bound proton pump that maintains the acid condition of the lysosome [54]. Injection with acidic solutions reduced cell pH which may have destabilised the lysosomal membrane, or the H+ ion pump, causing neutral red to leak into the cytosol of the cell, elevating our results. Our results suggest that the cellular processes of COTS have a low adaptive capacity following exposure to acidic injections [55] and may have resulted in their death. On the other hand, bacterial infection is also known to increase lysosome permeability (e.g. [56]) resulting in apoptosis [57]. Lysosomal membrane stability in the European flat oyster *Ostrea edulis*, Pacific oyster *Crassostera gigas*, scallop *Pecten maximus* and mussel *Mytilus galloprovincialis* was reduced after inoculation with bacteria [58, 59]. We also tested other aspects of the immune response and found elevated respiratory burst and peroxidase activity. Respiratory burst plays an important role in microbial activity and during phagocytosis, reactive oxygen species (ROS)-like superoxide anions (O_2_^−^), H_2_O_2_, and hydroxyl radicals (OH·) are produced [60]. Similar to our results, acidic pH stress induced respiratory burst in the Pacific white shrimp, *Litopenaeus vannamei* [61, 62]. Furthermore, exposure to acidic environments coupled with ROS production can act synergistically to cause extensive DNA damage leading to apoptosis [63, 64], which could explain death in the COTS we injected with acidic solutions. Peroxidase activity represents the activity of antimicrobial cytotoxic molecules and has a pH optima in the acid range [65]. Thus, increased activity after treatment with acidic injections is concordant with optima pH conditions in the acid range. However, ROS production and peroxidase activity also increase after bacterial infection in seastars (e.g. [66]) and shrimp [62].

Therefore, whilst our results cannot conclusively distinguish between whether lysosomal membrane permeabilisation and increase in peroxidase and respiratory burst were triggered by pH stress or a bacterial infection, we found that injection with artificial seawater also increased two of our three immune measures compared to those COTS receiving no injection. Cellular immunity takes place in the open coelomic system of COTS whose cavities contain coelomic fluid that is similar to seawater [67]. Artificial seawater contains no bacterial agents. Thus the slight increase in our immune measures could have been triggered by the small pH difference between the artificial seawater and the coelomic fluid of COTS, rather than bacterial infection. Furthermore, when coupled with the lack of disease transmission in the contagion experiments, the most parsimonious explanation for the death of COTS would indeed be the pH stress of the injections themselves, rather than an induction of a transmissible bacterial disease. Sub-lethal acute acid stress occurs at pH 5 and below [52] and as the pH of lime juice and vinegar was 1.8 and 2.2 respectively, the treatment injections likely represent chronic pH stress for COTS. Therefore, our multiple immune responses suggest that the COTS death was caused by pH stress.

### A new “cheap and natural” method to control COTS outbreaks

Our results show that acidic injections (namely lime juice or vinegar) offer an effective solution in controlling COTS outbreaks. This approach is particularly suitable for remote Pacific islands communities with limited financial resources. In Vanuatu where the corals ecosystems are mostly represented by narrow fringing reefs, COTS population can locally reach extremely high densities. Peak densities from 800 to 4 200 COTS.ha^-1^ were recently reported from surveys in six islands by Dumas et al. [68]. The injection scenarios tested in this paper demonstrated that 10–20 l of lime juice/vinegar was sufficient to kill a thousand COTS. While the limes themselves can eventually be provided at a nominal cost, or even for free by committed coastal communities, extracting such quantities of lime juice may still require significant manpower. Vinegar represents a more convenient, yet slightly more expensive alternative; it is ready to use and widely available from most local stores in the Pacific region. In Vanuatu, the average cost for one litre of vinegar is about 1.5 USD in the capital city; it would cost 15–30 USD to kill one thousand COTS, i.e. less than 0.05 USD per COTS, a noticeable difference compared to the cheapest current methods (e.g. 0.29 USD for bile salts). Moreover, our proposed control method uses 100% natural products which likely constitute safe, harmless alternatives to any of the other existing methods. No immediate or delayed effects were observed by reef fish, corals, and other benthic invertebrates, despite a high concentration of specimens in a limited water volume. While studies assessing malignant effects in the presence of injected COTS remain limited, this suggests that limited or no side-effects are likely in coral reef communities. The amount of acidic solution introduced into the marine ecosystem should be considered small enough not to interfere with the pH of seawater.

While this method is easily implementable under a community-based approach in shallow reef areas (e.g. < 10 m), COTS distribution may extend much deeper. Thus out of the reach of the local snorkelers. In the latter case, injections would need to be carried out by SCUBA divers, requiring extra resources from outside the community. Yet the manual collection methods currently used by most coastal communities share the same drawbacks, but require many more participants and time for a lower removal rate since the snorkelers have to make return trips to the boat [20]. The use of dehydrated acidic solutions also represents a promising alternative. Powdered citric acid is commonly employed in the food industry because of its preservative and chelating properties; it is available for purchase at low costs from a variety of stores. Acidic solutions extracted from other local acidic fruits may also be tested and used when lime and vinegar are not available (e.g. grapefruit, tangerine or natural hybrids of citrus).

More effectively controlling COTS outbreaks could potentially reverse the ongoing coral loss and reef degradation in the Indo-Pacific [14]. While injection methods are only short-term responses to a complex phenomenon whose ultimate causes are not fully understood, their efficiency is increasingly recognised as suitable for protecting isolated or individual reefs [69]. In this context, acidic injections of lime juice and vinegar offer great advantages when compared to current best practises and constitute a cheap and natural option for all countries affected by COTS.

## Supporting Information

S1 File Detailed methods for: Mechanistic basis for death from acidic solutions. (DOCX)
