# Supplementary material for: Lime Juice and Vinegar Injections as a Cheap and Natural Alternative to Control COTS Outbreaks
Source: PLoS One. 2015 Sep 10;10(9):e0137605. doi: 10.1371/journal.pone.0137605 (PMC4565713; doi:10.1371/journal.pone.0137605)
Supplement: S1 File — (DOCX) [file pone.0137605.s001.docx]

**Detailed methods for: Mechanistic basis for death from acidic solutions**

**Immune response solutions**

Calcium and magnesium-free artificial seawater (CMFASW) was made up by adding 28.333 g NaCl, 0.665 g KCl, 3.916 g Na_2_SO_4_ and 0.194 g NaHCO_3_ to 1 l Nanopure water. Antiaggregative solution (AG) was made up of CMFASW containing 15 mM ethylenediamine tetracetic acid (EDTA).

**Baseline (pre-treatment) immune response and coelomic fluid collection**

Within three hours of collection from the fringing reef, a subset of individual *A. planci* were blotted with a sorbing paper and 1.5 ml of coelomic fluid (CF) was withdrawn into an equal volume of cold AG by inserting a 3 ml pre-cooled syringe fitted with a sterile 23 gauge needle into the distal third of one arm. The resulting CF-AG suspension was homogenised immediately to prevent coagulation, thrombus formation and secretory degranulation of coelomocytes. The CF-AG suspension was then subdivided into three sub-samples in order to measure three aspects of immune function described below.

**Post-treatment immune response**

To simulate bacterial invasion, 1 ml of a suspension of the dead Gram-positive bacteria *Micrococcus lysodeikticus* (10 mg ml^-1^, Sigma M0508) in 0.1 M NaHCO_3_ buffer, pH 9.0, was injected into each *A. planci* (approx. 0.06 ml per arm) [37] 5.5 days prior to acidic treatment injections. At 1600 hrs on the fifth day individual COTS received one of four treatments at random (no injection or 10 ml injection of either artificial seawater, vinegar or lime juice). Coelomic fluid was collected as described above 6 d post-bacterial challenge [36].

**Lysosomal membrane integrity**

The neutral red (NR) assay is a measure of an individual’s response to stress via the retention of a neutral red dye in lysosomes [40]. In unstressed cells, lysosomes will accumulate and retain neutral red dye for an extended period of time; however, once destabilized by a stress response the neutral red dye will leak into the cytosol of the cell through the damaged lysosomal membrane [38, 39]. Lysosomal integrity of amoebocytes was evaluated using the method of uptake of the cationic probe NR as adapted from Bekri and Pelletier [37] and for *A. planci* [36]. 0.1 ml of NR (Acros Organics 415490250) was added to 1 ml of CF-AG suspension. The samples were incubated in the dark at 18 °C for 75 min. After centrifugation at 552 *g* for 5 min, the supernatant was removed and fixed in 400 µl of 5% formaldehyde in CMFASW during 15 min with mild manual shaking. After centrifugation, the fixative solution was removed and replaced by 1 ml CMFASW, and the preparation was stored in the dark at 4 °C. Prior to the reading day, the supernatant was replaced by 1 ml of the extraction solution containing 1% (v/v) acetic acid + 50% (v/v) ethanol in Nanopure water, and cells were incubated for 15 min at room temperature with occasional shaking to release neutral red retained by lysosomes. The absorbance of neutral red extract was measured at 490 nm on a BioTek Microplate Reader ELx800UV (BioTek Instruments GmbH, Bad Friedrichshall, Germany) calibrated with the extraction solution. Results are expressed as units of optical density (OD) per well.

**Oxygen metabolism of coelomocytes**

This test determines if immune system cells can change the colourless chemical nitroblue tetrazolium (NBT) into a deep blue colour and follows the method described by Mills (2012). 1.0 ml of CF-AG suspension was softly centrifuged at 100 *g* for 10 min. The supernatant was removed and 400 µl of CMFASW added during 15 min with mild manual shaking. 100 µl of the cell suspension was placed into a 96-well flat-bottom microtiter plate (Fisher Scientific, Strasbourg) in duplicate. To determine the induced index of the NBT-test, 50 µl of 0.05% suspension of Zymosan A from *Saccharomyces cerevisiae* (Sigma, Z4250, Analytic Lab, St Mathieu de Treviers) in CMFASW was added. 50 µl of 0.2% *p-*NBT (Sigma, 84010, Analytic Lab, St Mathieu de Treviers) were added into all wells. The plates were incubated for 24 h at 26 °C (local seawater temperature). The supernatant was removed, thereby terminating the reaction. To fix the samples, 50 µl of 95% ethanol (Fisher Scientific, M0346X) was added into each well. The plates were placed in a Diemos Fisherbrand thermostat at 37 °C for 6-8 h until complete drying. The plates were stored at -20 °C prior to spectrophotometric recording. On the reading day, the plates were defrosted, and 200 µl of distilled water was added into each well and the plates were centrifuged at 400 *g* for 7 min. The supernatant was removed and 120 µl of 2 M KOH (Sigma, P5958, Analytic Lab, St Mathieu de Treviers) in water and 140 µl of dimethylsulfoxide (Sigma D8418, Analytic Lab, St Mathieu de Treviers) were added into each well. Complete dissolution of NBT diformazan granules was achieved by incubation of the plate on a shaker at 37 °C for 1 h. The plates were read at 630 nm on a BioTek Microplate Reader ELx800UV (BioTek Instruments GmbH, Bad Friedrichshall, Germany). Results were expressed as units of optical density (OD) per well.

**Peroxidase activity**

50 µl of CF-AG suspension was added into wells of a 96-well flat-bottom microtiter plate (Fisher Scientific, Strasbourg) in duplicate. As a control, 50 µl of CMFASW was added. 50 µl of the chromogenic mixture containing 0.5% orthophenylenediamine (Sigma, P23938, Analytic Lab, St Mathieu de Treviers) in phosphate-citrate buffer, pH 5.0 (Sigma, P4809, Analytic Lab, St Mathieu de Treviers), and 0.01% hydrogen peroxide (Sigma, 216763, Analytic Lab, St Mathieu de Treviers) were added into each well. The samples were incubated in darkness in a Diemos Fisherbrand thermostat at 37 °C for 75 min. The reaction was terminated by the addition of 50 µl of 2 M sulfuric acid. Intensity of the reaction was determined at 490 nm using a BioTek Microplate Reader ELx800UV (BioTek Instruments GmbH, Bad Friedrichshall, Germany). Results were expressed as units of optical density (OD) per well. Baseline measures of peroxidase activity were not taken in the second trial due to a lack of sulphuric acid, which was preferentially conserved for post-treatment measures.
